# Unsupervised analysis of NIRS spectra to assess complex plant traits: leaf senescence as a use case

**DOI:** 10.1186/s13007-022-00927-6

**Published:** 2022-08-12

**Authors:** Héloïse Villesseche, Martin Ecarnot, Elsa Ballini, Ryad Bendoula, Nathalie Gorretta, Pierre Roumet

**Affiliations:** 1 AGAP, CIRAD, INRAE, Institut Agro, Univ Montpellier, Montpellier, France; 2 PHIM, CIRAD, INRAE, IRD, Institut Agro, Univ Montpellier, Montpellier, France; 3 ITAP, INRAE, Institut Agro, Univ Montpellier, Montpellier, France

**Keywords:** NIR, Leaf senescence, Unsupervised model, Wheat, Temporal kinetic

## Abstract

**Background:**

As a rapid and non-destructive method, Near Infrared Spectroscopy is classically proposed to assess plant traits in many scientific fields, to observe enlarged genotype panels and to document the temporal kinetic of some biological processes. Most often, supervised models are used. The signal is calibrated thanks to reference measurements, and dedicated models are generated to predict biological traits. An alternative unsupervised approach considers the whole spectra information in order to point out various matrix changes. Although more generic, and faster to implement, as it does not require a reference data set, this latter approach is rarely used to document biological processes, and does requires more information of the process.

**Methods:**

In our work, an unsupervised model was used to document the flag leaf senescence of durum wheat (*Triticum turgidum durum*). Leaf spectra changes were observed using Moving Window Principal Component Analysis (MWPCA). The dates related to earlier and later spectra changes were compared to two key points on the senescence time course: senescence onset (T0) and the end of the leaf span (T1) derived from a supervised strategy.

**Results:**

For almost all leaves and whatever the signal pre-treatments and window size considered, the MWPCA found significant spectral changes. The latter was highly correlated with T1 (0.59 ≤ r ≤ 0.86) whereas the correlations between the first significant spectrum changes and T0 were lower (0.09 ≤ r ≤ 0.56). These different relationships are discussed below since they define the potential as well as the limitations of MWPCA to model biological processes.

**Conclusion:**

Overall, our study demonstrates that the information contained in the spectra can be used when applying an unsupervised method, here the MWPCA, to characterize a complex biological phenomenon such leaf senescence. It also means that using whole spectra may be relevant in agriculture and plant biology.

**Supplementary Information:**

The online version contains supplementary material available at 10.1186/s13007-022-00927-6.

## Background

The plant phenotype, i.e., the set of observable characteristics of an individual, reflects both the gene expression (genotype) and the amount of available resources (mineral nutriments, water, temperature, light) as well their interaction. Phenotypic measurements are essential to understand how organisms interact with their environments to identify, for example, the genetic determinism involved in local adaptation, or to achieve a more sustainable production in agronomy. Too often, measurements of most of these traits require destructive and time costly methods, limiting extended observations of numerous individuals and/or along temporal series. The development of methodologies to assess an enlarged list of these traits is of great interest for many fields of study: agronomy, ecology, genetics and phytopathology. For two decades, numerous applications based on non-invasive and non-destructive methodologies, such as imaging analysis or near infrared spectroscopy, have been developed to infer trait values [1, 2]. As most of them are complex traits resulting from numerous and successive processes as well as their interactions, two main complementary strategies have been proposed to phenotype these traits.

The first one leads to the dissection of these complex traits into elementary traits to facilitate the identification of underlying physiological pathways or genetic determinism. This strategy is commonly used in scientific communities such as physiologists or geneticists for whom a better understanding of the building of complex traits is essential. As most often the elementary traits are not easily visually observable, extensive works related to the use of some non-destructive and non-invasive methods, largely based on the use of visible and near infrared (VIS–NIR) imaging and spectroscopy, have been undertaken [3]. Thanks to supervised models based on linear or nonlinear signal treatment methods, the calibrations provided relevant predictions with documented accurateness and robustness which can be used as proxies of the key traits targeted [4, 5]. The second strategy directly targets the complex trait to document some changes, or to detect some possible discrepancies along its time course; this global approach is frequently used in a wide range of disciplines such as transformation process monitoring, agronomy and phytopathology [6]. Both strategies are largely supported by the development of high throughput phenotyping platforms.

Overall, if rapid and non-destructive phenotyping methods such as VIS–NIR spectroscopy are highly desirable to document some changes in traits during their time course, both supervised and unsupervised methods—which do not require any signal calibration- can be used. Indeed, according to the hypothesis that a VIS–NIR spectra signal is related to the global status of the biological matrix at a given time, non-supervised models based on raw spectra information gathered along the process are pertinent when documenting some matrix changes during its time course, providing some alerts [7]. Different unsupervised methods have been proposed to document matrix changes but all follow the same principle: no calibration is made on the data set. The idea is to find common characteristics between spectra and to find hidden patterns indicating the presence or absence of change in the matrix. Most of the unsupervised strategies are based on the Principal Component Analysis (PCA). Among them, Moving Window Principal Components Analysis (MWPCA) has been developed to accurately pinpoint small changes in process monitoring [8].

Leaf senescence, which is the final stage of leaf development, is one example of these complex biological processes. Besides chlorophyll degradation which is well known, numerous additional molecular and biochemical processes contribute to the senescence syndrome [9, 10]. When a leaf switches its developmental program from maturation to senescence, many leaf mobile nutrients involved in different pathways, such as carbon acquisition, are degraded and recycled for re-use in upper and still growing parts of the plant, e.g. younger leaves or in the forming reproductive organs. As the content of many biochemical components drop significantly during senescence [11], this process can be documented through many traits: leaf green area losses, leaf water content, decline of photosynthetic capacities [12] or a decrease of leaf biochemical content such as chlorophyll, nitrogen or water [13]. Whatever the trait considered, the senescence process follows a similar temporal pattern: after a lag phase during which the leaf maintains its physiological activity at a constant level, the content of biochemical components or enzymatic activity drops dramatically to reach a second plateau with minimal values. On cereals and particularly on wheat the timing of the onset and the end of senescence are considered as key points when comparing genotype senescence properties [14–16], as well as targeted physiological traits to predict grain yield since positive correlations have been reported between delayed senescence and grain yield both under limiting and non-limiting conditions [17, 18].

Based on spectra reflectance, numerous supervised models have been extensively used to generate some proxies of these traits to facilitate the collection of phenotype data sets. However these components have different time courses and they interact with the amount of resources available (nutrients and water in particular), demonstrating different senescence dynamics and an environmental dependent response [5]. These studies have shown that there is an intra specific variability in the onset and rate of senescence (for example see [16, 19, 20]. The onset of senescence is a complex trait that illustrates the transition of the leaf when grain N demand exceeds soil N uptake, generating translocation of N from stems and leaves and affecting carbon assimilation [21]. A delay in this onset or a lower rate of senescence will have a direct impact on wheat yield [5, 22–24].

The idea that the spectral signature could be used directly to characterize the presence and the state of vegetation was proposed following the development of the early sensors which had low spectral resolution [18, 25]. The first use of a spectral index, the normalized difference vegetation index (NDVI) was described by Rouse et al. [11] to document vegetation in the central region of the United States. Nevertheless, important limitations were reported for the use of NDVI to document the leaf senescence process. Only two wavelengths have been used to compute the index, NDVI is focused on chlorophyll content, which turned out to be non-relevant to physiological changes occurring during senescence onset [26]. To avoid some confounding effects and to properly assess leaf senescence, the use of a higher number of bands made possible by a better spectral resolution device coupled with a supervised model was proposed [4]: visual senescence scoring was used as reference measurements and calibration of spectra data was performed to fit the senescence dynamics during the grain filling period. Here, the use of the supervised approach can be debated. Firstly, the reference analysis was based on visual information; as this measurement was neither objective nor precise, the generated background noise affected the accuracy of the model; secondly, the validity domain of this calibration is uncertain, thus limiting its use to new data sets. Thus, regarding the complexity of the senescence process and the limits of supervised models, an alternative approach based on an unsupervised model such as MWPCA where it is postulated that some changes occurring over time in a spectral data set are a signature of the senescence process seems promising.

In order to establish the limits of the unsupervised approach, we have compared the time course of flag wheat leaf senescence using both supervised and unsupervised models based on spectral reflectance. Large range of senescence time courses was generated thanks to the genotype sampling and mineral nutrition availability.

## Materials and methods

### Plant material

A total of 113 F_8_ Recombinant Inbred Lines (RILs) from six crosses involving four durum wheat cultivars (Néodur, Primadur, Lloyd and Ixos) were used in the present study since they represented a high variability for senescence time course parameters.

### Experimental conditions

The trial has been conducted on durum wheat (*Triticum turgidum durum*) in greenhouse at the DiasCope experimental unit from Institut National de Recherche pour l'Agriculture, l'Alimentation et l'Environnement (INRAe, Mauguio, France, 43°37′ N, 4° E) in 2011Plants were sown on 5 Jan. 2011 in Jiffy peat pellets. After vernalization in a controlled chamber (4 °C for 5 weeks) they were transferred to a greenhouse (15 Feb. 2011) and grown in vermiculite in 1.2 L pots (one plant per pot). To maximize the variability range of flag leaf senescence,plants have been cultivated under two types of nitrogen fertilization conditions and observed daily from flowering to maturity. In the first nitrogen treatment, 104 plants (one plant/RILs), were irrigated once daily with 5 mM KNO3 until anthesis and with 1 mM KNO3 from anthesis to grain maturity; in the second one, 35 plants (representing 35 different RILs) were irrigated once daily with 1 mM KNO3 until anthesis and then with 5 mM KNO3 for 550 Growing Degree-Day -GDD- after heading. Potassium concentrations were adjusted for treatments with 1 mM KNO3 by adding 4 mM KCl to the nutrient solution. Irrigation was stopped when the grain reached physiological maturity. Greenhouse temperature was regulated by a cooling system and the opening and closing of greenhouse vents and was measured with independent sensors. The walls of the greenhouse were whitewashed to standardize the amount of light received by each plant. Further information about the settings of the experiment are described in Vilmus et al. [27].

### Spectra acquisitions

For each plant, all observations were made daily on the last leaf (flag leaf) from flowering to physiological maturity (stages Z65 and Z85 from Zadoks scale); they were numbered from one (for the first at flowering) to n (the last at physiological maturity). Observations were made in a non-destructive manner with a LabSpec 2500 portable Vis/Nir spectrometer, which has a spectral range between 350 and 2500 nm with a spectral resolution of 3 nm between 350 and 1000 nm to 10 nm between 1000 and 2500 nm (Analytical Spectral Devices, Inc., Boulder, CO, USA) using the LeafClip and a white background panel, allowing us to obtain transflectance spectra [28]. Each day, two measurements were performed by dividing the flag leaf into three equal parts along its length, with the first measurement made on the proximal third and the second on the distal third.

Based on these spectra data two different analysis were performed; firstly, a classical supervised strategy was used: calibrations were made to document the biochemical properties of the flag leaf during senescence process; secondly, whole spectra information (raw or pre-processed spectra data) were used in an unsupervised strategy to document the evolution of the leaf main features.

### Supervised strategy: NIR calibration development and temporal kinetic modeling

In a first step, quantitative calibrations from whole spectra were made to assess three biochemical leaf parameters involved in leaf senescence from data spectra, namely nitrogen, chlorophyll and water content. To carry out these calibrations, a small but representative group of samples were selected to build the models, and these calibrations were applied on all other measured spectra. A common protocol was applied for the three calibrations. First, spectra were taken, as described above, on leaves that were analyzed with a specific reference method for each parameter. Then calibrations were performed with Partial Least Square regression [29], using random repeated k-fold cross-validation to optimize the number of latent variables of the model, and to compute model-fitting statistics (standard error of cross validation, coefficient of determination). For more details, please refer to the Additional file 1: Table S1.

In a second step, logistic regression was used to fit the temporal evolution of these components from flowering to the physiological maturity where time was expressed in cumulative GDD based on average daily temperature. For each leaf, the model is estimated as in Eq. (1):

1 $$ f\left( x \right) = d + \frac{a}{{1 + b*e^{c*x} }} $$

where a, b, c and d are parameters allowing the adjustment of the sigmoid curve for each individual and x being the date given in GDD. The results extracted from the model are the T0 and T1, corresponding respectively to the end of the first plateau and the end of the drop of the sigmoid curve. Here, these two key points have been considered as the onset and the end of the senescence. For each flag leaf, T0 and T1 have been computed for each of the three types of biochemical components according to the following formulae:

2 $$ T_{0} = d + \frac{{a\left( {1 - \gamma } \right)}}{{1 + e^{b} }}\,\,\,{\text{and}}\,\,T_{1} = d + \frac{a\gamma }{{1 + e^{b} }} $$

where a, b and d are three parameters of the sigmoid curve (Eq. 1) and $\gamma$ =0.01.

Model evaluation was made using the efficiency calculated as in Eq. 3:

3 $$ Efficience = 1\, - \frac{{\mathop \sum \nolimits_{1}^{i} \left( {y{||}i - f\left( {x_{i} } \right)} \right)}}{{\mathop \sum \nolimits_{1}^{i} \left( {y{||}i - mean\left( y \right)} \right)}} $$

where i is varying from 1 to the number of dates taken in the time-series for each individual and y is the result of f(x). This model is described in detail in Vilmus et al. [27].

### Unsupervised strategy: Moving Window Principal Component Analysis (MWPCA)

A MWPCA [8] based approach was proposed here to detect changes that could lead the process to dysfunction, and then give an early alarm. Spectra collected at the successive dates from flowering to physiological maturity represented data variation. An abnormal deviation of spectra from those collected before could be considered as a major change in leaf features. To begin, Principal Component Analysis (PCA) was calibrated on training data representing the initial leaf state. From this model, the covariance and loading matrix P were computed. Secondly, new observations were projected onto the reference PCA model; a T^2^ metric was used to quantify spectra changes at t + 1. The new observation xi is centered, using the parameters of the training data.

The initial PCA model was calibrated for each leaf, considering a spectra data matrix X (n × p) with n the number of observations and p the number of spectral variables (Eq. 4):

4 $${X}_{(n\times p)}={T}_{(n\times a)}\cdot {P}_{(a\times p)}^{T}+{E}_{(n\times p)}$$

T is the score’s matrix with a (a = 1, 2 …) the number of retained Principal Components (PCs), P^T^ is the transpose of loadings matrix which explains the relationship between spectral variables and PCs and E is the residual matrix. Before implementing PCA, variables are centered. PCA control charts are based on the Hotelling’s T^2^ statistics, being the squared Mahalanobis distance of the spectra x in the PCA model subspace [8] and calculated according to Eq. 5:

5 $${T}^{2}={x}_{i}{P\Lambda }_{a}^{-1}{P}^{T}{x}_{i}^{T}$$

where ∧ is the covariance matrix of the a scores column and xi is the i-th observation predicted through the PCA model. To define the change in the time-series, the hypothesis is made that the Hotelling’s T^2^ is following a normal law. Based on this hypothesis, a significant change is supposed to be a T^2^ out of the normal distribution, modeled by the normal law. Significant changes will not be excluded from the next window, because this “new state” of the process will affect the next observations and could even result in a new normal steady state.

**Fig. 1:**
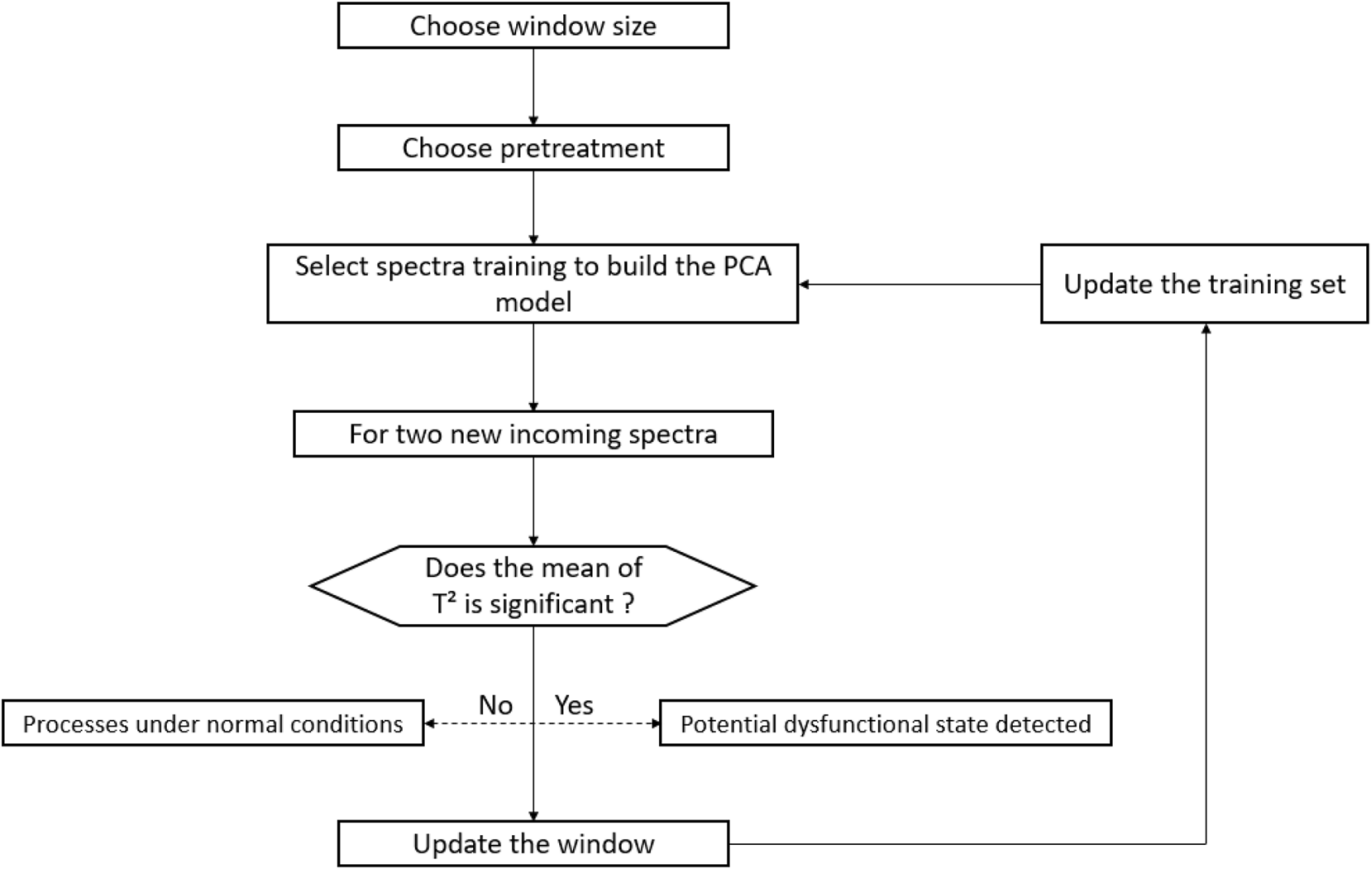
Moving window PCA algorithm (adapted from by Awhangbo et al. [7])

The adopted algorithm of the MWPCA is shown in detail in Fig. 1.

In MWPCA, the basic algorithm stipulates that a data window of fixed length be moved in real time to update the PCA model once a new sample is available. With each new observation, this window excludes the oldest observation and includes the newest one. One challenge in implementing MWPCA is to select the window length (also called window size). Due to the number of our dataset observations (~ 30 dates), we decided to test the influence of the window size on our dataset. During the analysis, windows sized between 3 and 15 were tested, equivalent to taking from 3 to 15measurements to build the core spectra to refer in the initial foliar stage. The number of components was also updated to correspond to the maximum number of components possible according to the window size. Knowing that two spectra of each plant were taken every day, the maximum number of components corresponds to twice the window size minus one.

Based on the preprocessing, three non-supervised analyses were carried out. The first was based on raw spectra data and called ‘no pretreatment’ while the two others used the two most common preprocessing: Standard Normal Variate (SNV, [30]) method and a combination of SNV and the Savitzky-Golay filter (SAVGOL, [31]) which can also be found in the construction of the calibration model made by Ecarnot et al. [28]. The SAVGOL filter was used with a window of 15 nm and a first-degree derivative with a polynomial of the second degree. In total three parameters have varied during the analysis: the window size, the number of components and spectra preprocessing.

In this work, spectra collected at the successive dates from flowering to physiological maturity represented the varying data. MWPCA based approach was proposed here to detect changes that could lead the process to dysfunction and then give an early warning. An abnormal deviation of spectra from those collected before could be considered as an out-of-control state. To define the change in the time-series, we made the hypothesis that Hotelling's T^2^ followed a normal law. The 2.5% higher T^2^ values were considered out of the normal distribution, and representative of a new steady state in the leaf senescence process. Significant changes will not be excluded from the next window contrary to the usual algorithm, because this “new state” of the process will affect the next observations and could even result in a new normal steady state. To compare the results from MWPCA to those from the targeted strategy, the day numbers for which spectra generated significant values of T^2^, named T^2^ peak, were converted into cumulative GDD. All the analyses were performed with R software [32].

## Results

### Spectra data and leaf senescence process

**Fig. 2:**
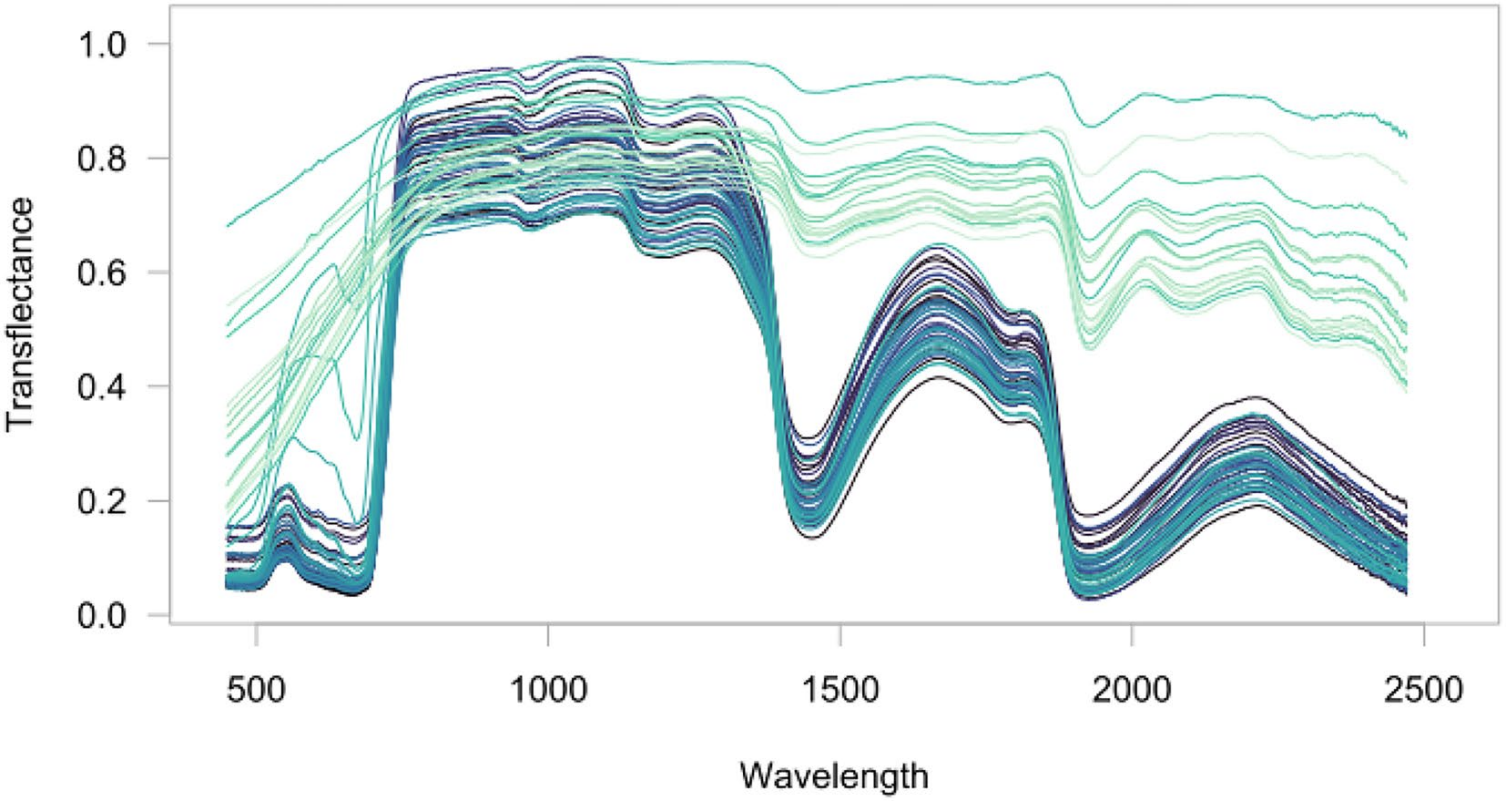
Raw spectra (from 400 to 2500 nm) of one individual leaf during senescence time course; the darkest blue curves correspond to the first date (flowering) in the experiment while the lightest blue curves indicates the last day of the experiment (end of leaf senescence)

Typical transflectance spectra of fresh leaves during post the flowering period have been plotted in Fig. 2. The effect of senescence is mainly perceptible in the visible region (around 690 nm), where the continuous degradation of chlorophyll generates a filling of the absorption trough, until it disappears for senescent leaves. The process is also acting in the short wave infrared (SWIR) region (1000–2500 nm) principally shaped by -OH bounds absorption, and mostly water. Indeed, the two most intense water absorption bands are located at 1450 and 1950 nm, and generate troughs in the transflectance spectra, especially for fresh leaves, which then get weaker for senescent leaves. Conversely, the 750–1000 nm spectra region, in which almost no absorption occurs, maintains the same average values all along leaf senescence. Along with the influence of biochemical compounds [33], the physical structure of the leaf determines the average value of the spectra. Leaves with high density have compact mesophylls leading to low scattering, and thus lower average transflectance, which explains the high variance in spectra averages of our data set.

### Supervised models of Nitrogen, Chlorophyll and Water leaf-content prediction

**Fig. 3:**
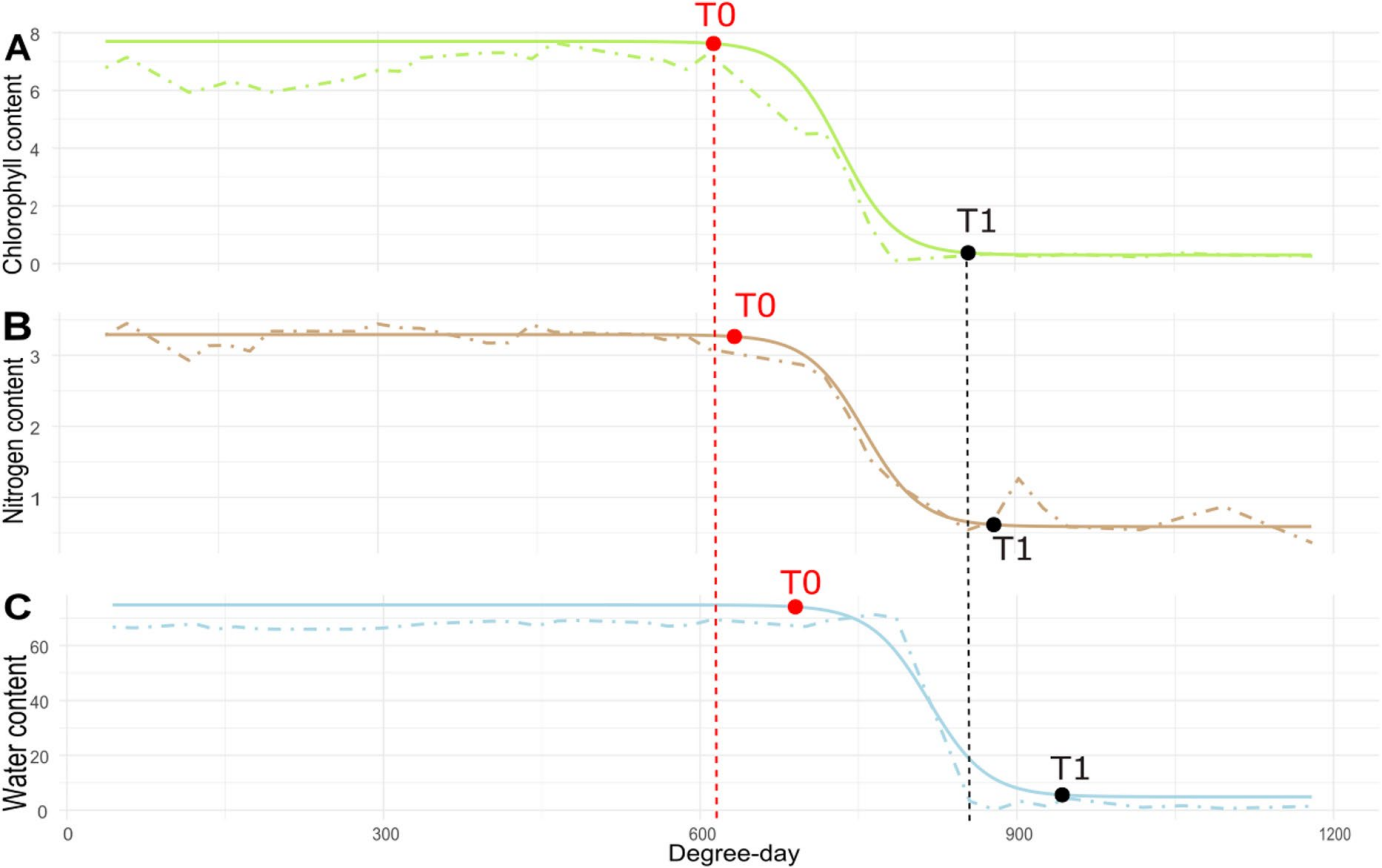
Time course of chlorophyll content (**A**) nitrogen content (**B**) and water content (**C**) of an individual flag leaf from flowering to maturity. The dash-dot lines represent the experimental date issued from NIRS calibration while the full lines result from the logistic curve modelled for each leaf trait. The red and black dots correspond to the T0 and T1 of each kinetic

The same pattern, a plateau/drop/plateau sequence, was observed (Fig. 3) for the temporal kinetics of the three leaf biochemical components targeted. Logistic adjustments were highly relevant to report these temporal sequences since the efficiency parameters were close to 1 (> 0.95). Based on these curves, two key points, T0 and T1 have been computed for leaf nitrogen, chlorophyll and water content (see Eq. 2). Their average values, as well as their variability for the 139 flag leaves, are reported in Table 1.

**Table 1:** Means and standard deviations (n = 139) of T0 and T1 derived from modelling of nitrogen, water and chlorophyll leaf content during the senescence process (accumulated growing GDD post anthesis)

Water content T_0_	Nitrogen content T_0_	Chlorophyll content T_0_	Water content T_1_	Nitrogen content T_1_	Chlorophyll content T_1_
659 ± 100	530 ± 118	505 ± 124	907 ± 129	916 ± 151	872 ± 134

Overall, the results show that chlorophyll content was the first to decrease, followed by nitrogen and water contents. The pattern changes for point T1: here the chlorophyll content also ends its variation first, which is followed by water then nitrogen. The standard deviation shows that all the distributions overlap each other, hence the need to treat each individual separately (Fig. 3).

### Unsupervised approach (MWPCA): spectra changes during the senescence process

Based on significant spectra information changes, peak of T^2^ values had been detected for most of the individuals (> 89%). The lowest values were obtained from coupling raw spectra data with a small learning window size (< 5 days) whereas the other combinations provided higher and similar proportions (from 94 to 99%, Table 2).

**Table 2:** Percentage of individuals with at least one significant T^2^ value over the 139 individuals

Spectra pre-treatment	Learning window size (number of daily spectra acquisitions)					
	3	4	5	6	7	8
SNV + Savgol	97.12	96.40	97.12	97.84	96.40	94.96
SNV	97.84	97.12	99.28	97.84	99.28	98.56
Raw spectra	89.93	89.93	97.84	93.53	97.12	97.84

**Fig. 4:**
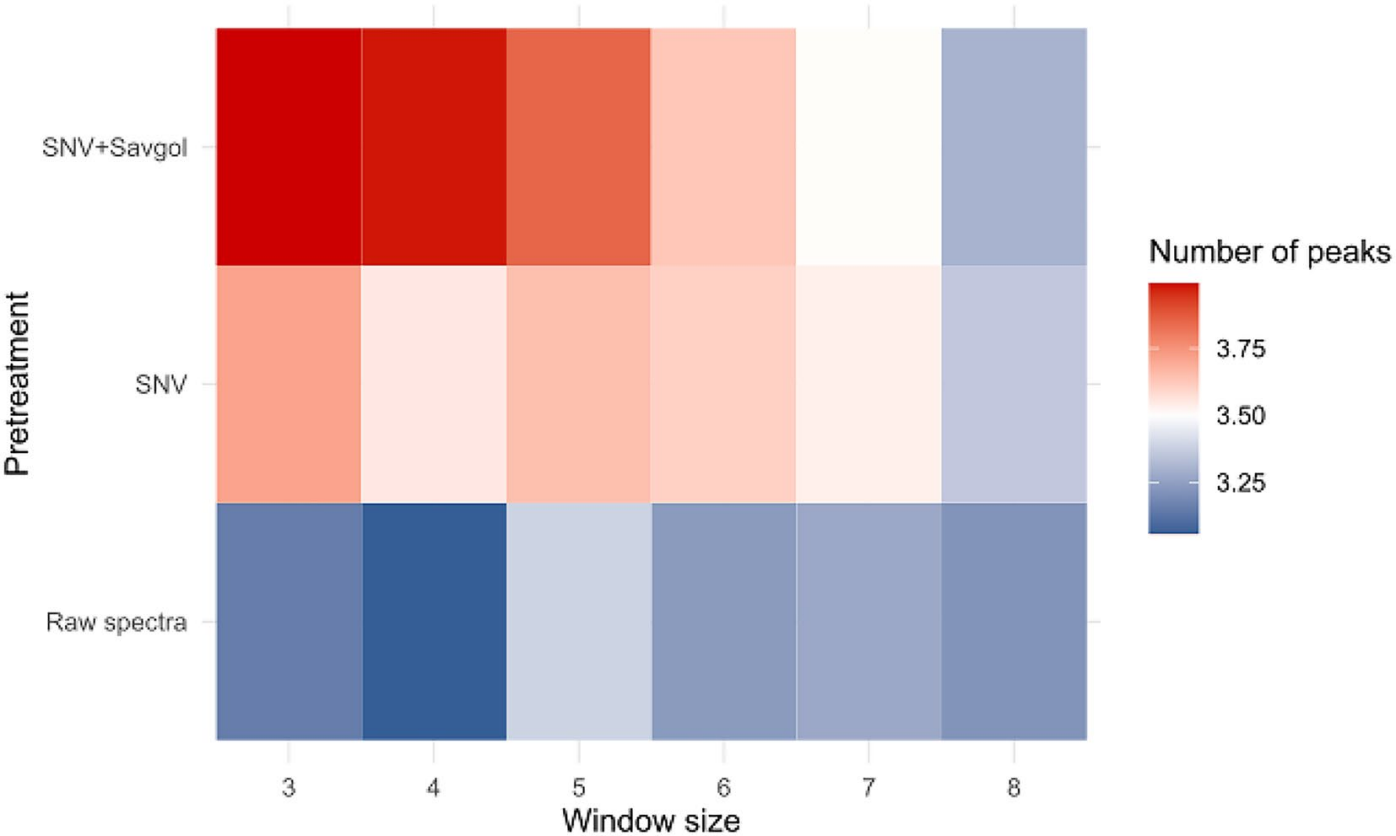
Number of T^2^ peaks found in the MWPCA according to the window size expressed in the number of daily spectra included in the reference spectra data set, and the spectra pretreatment

In addition, on average the number of significant T^2^ values (T^2^ peaks) detected per individual varied from 3 to 4 according to the learning window size and spectra treatment (Fig. 4). This T^2^ peak number was lowest with the raw spectra (about 3.22) and increased according to the pre-treatment process (3.57 and 3.71 for SNV and SNV + Savgol preprocessing respectively). Moreover, if the number of T^2^ peaks observed remained constant whatever the window size with raw spectra information, it decreased as the window size increased for the 2 others spectra preprocessing categories (3.36 < T^2^ < 3.71 and 3.30 < T^2^ < 4.00 for SNV and SNV + Savgol preprocessing respectively).

The information extracted from the MWPCA was condensed in the time interval between onset and end of senescence. To have a better understanding of the information resulting from the two strategies (supervised and unsupervised), we compared the timing given by the T^2^ peaks from MWPCA with T0 and T1 values from supervised modeling which are the signatures of the onset and the end of the leaf senescence process.

**Fig. 5:**
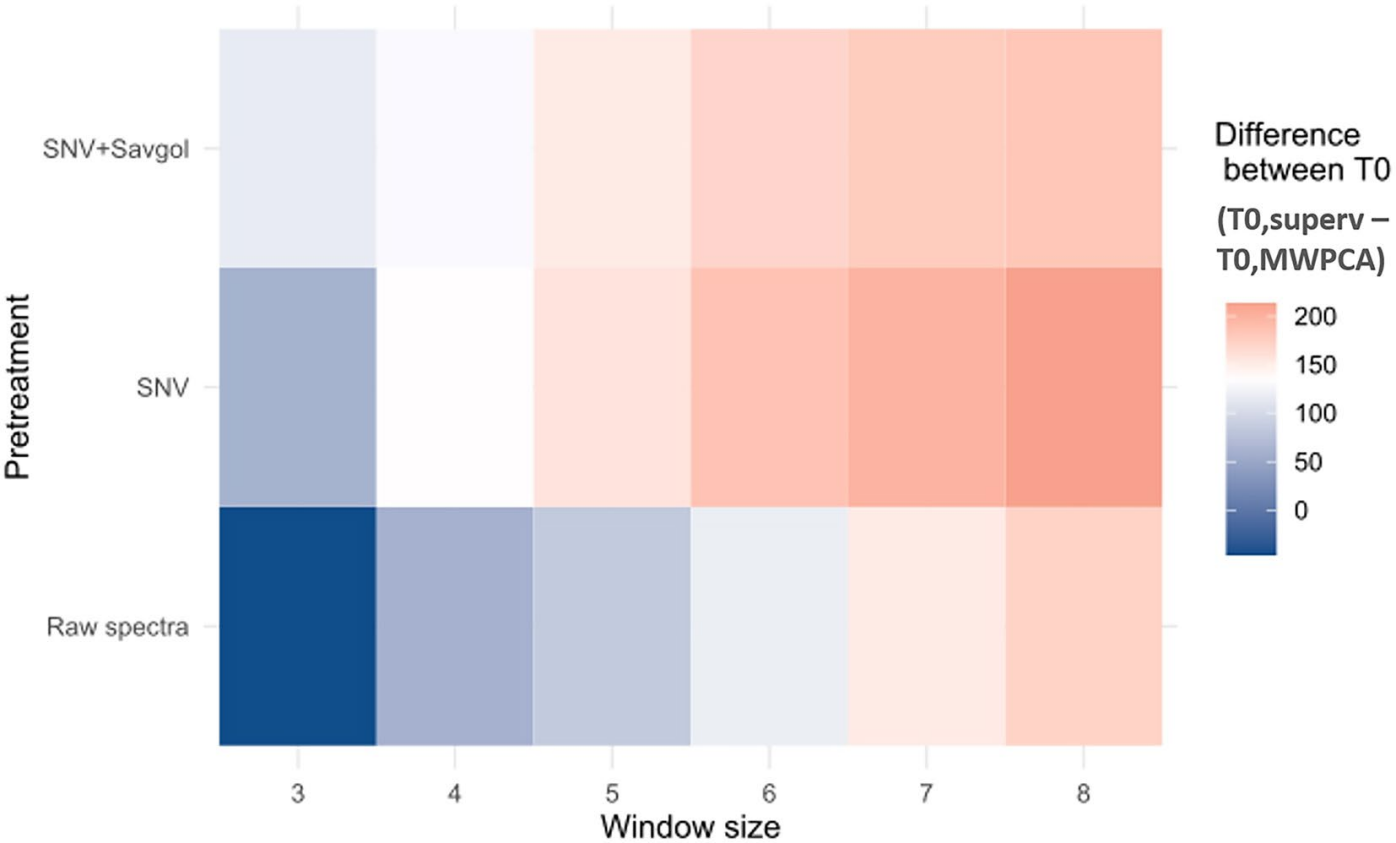
Difference expressed in GDD between the T0, superv and the T0, MWPCA according to the window size and the pretreatment

Firstly, we linked the earliest peak provided by MWPCA (T0,MWPCA) with the earliest T0 value observed among the three T0 values available (water, chlorophyll or nitrogen) (T0,superv) (Fig. 5). The difference and the correlation between these 2 variables T0, superv and T0,MWPCA were both considered.

Whatever the spectra preprocessing and window size considered, the first peak provided by MWPCA is later than T0, superv, which means that the MWPCA signaled a change in the system after the onset of the senescence (Fig. 5). The range of the differences depended on both pre-treatment and window size: minimum values were obtained with raw spectra and the lowest size window (between -47 and 85 GDD for a window size between 3 and 5). Whatever the spectra pre-treatment, increasing the learning window size amplified these differences (up to 171 GDD).

**Fig. 6:**
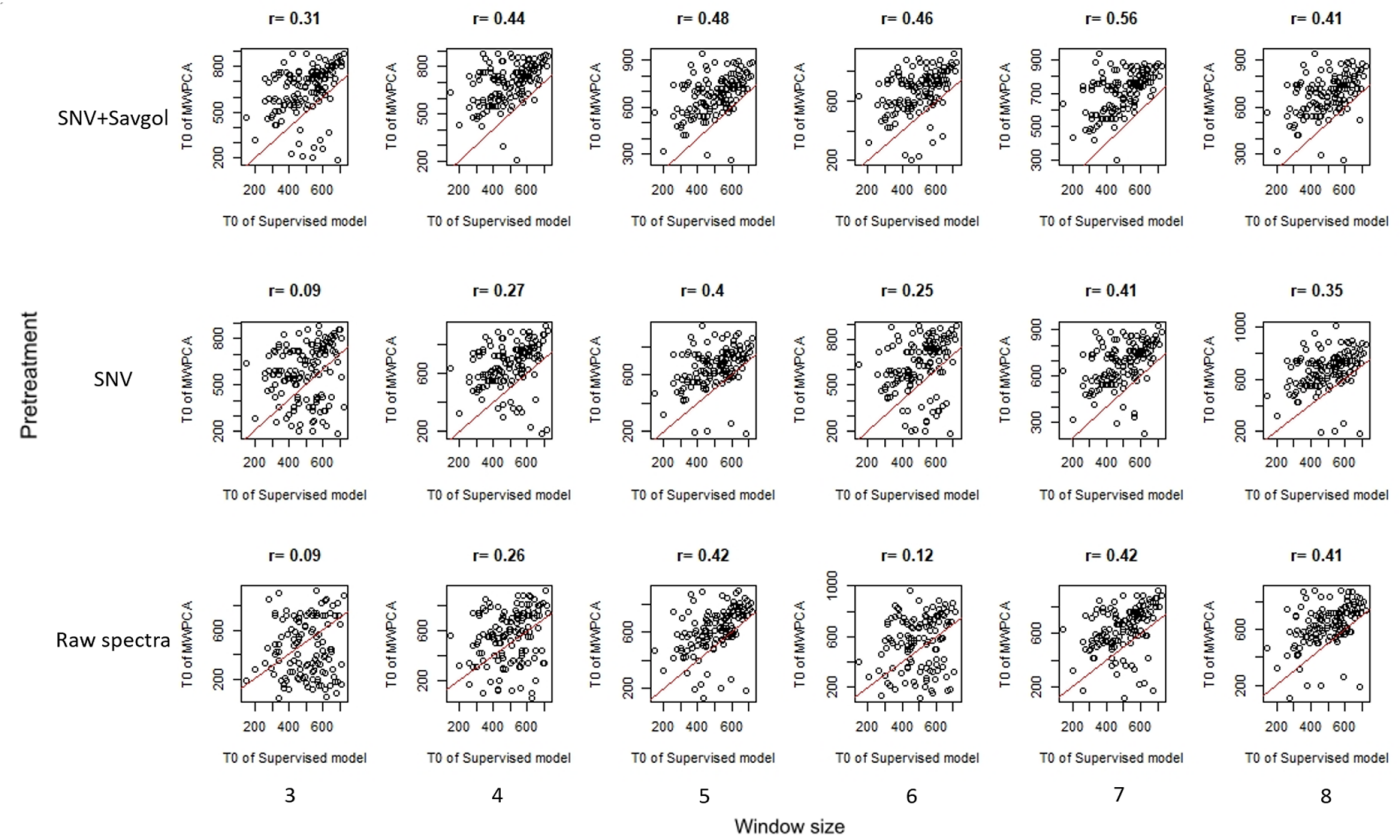
Relationship between T0, superv and T0, MWPCA. The red line corresponds to the 1:1 regression line and the number above each distribution to the correlation between the T0, superv and the T0, MWPCA

Furthermore the correlations between T0, MWPCA and T0,superv were lowest when the differences between the 2 indicators were minimum (raw or SNV spectra coupled with a small learning window size, Fig. 6, r = 0.09). For these two spectra pre-treatments, the correlation values increased according to the size of the window to reach similar values, whereas for the SNV-Savgol pre-treatment, the correlation remained stable whatever the window size considered. Thus, if similar correlations were obtained in the three pre-treatment with a large window size, it is noticeable that a more significant spectra pre-treatment provided correlations between T0,MWPCA and T0,superv that were less related to the size of the window.

**Fig. 7:**
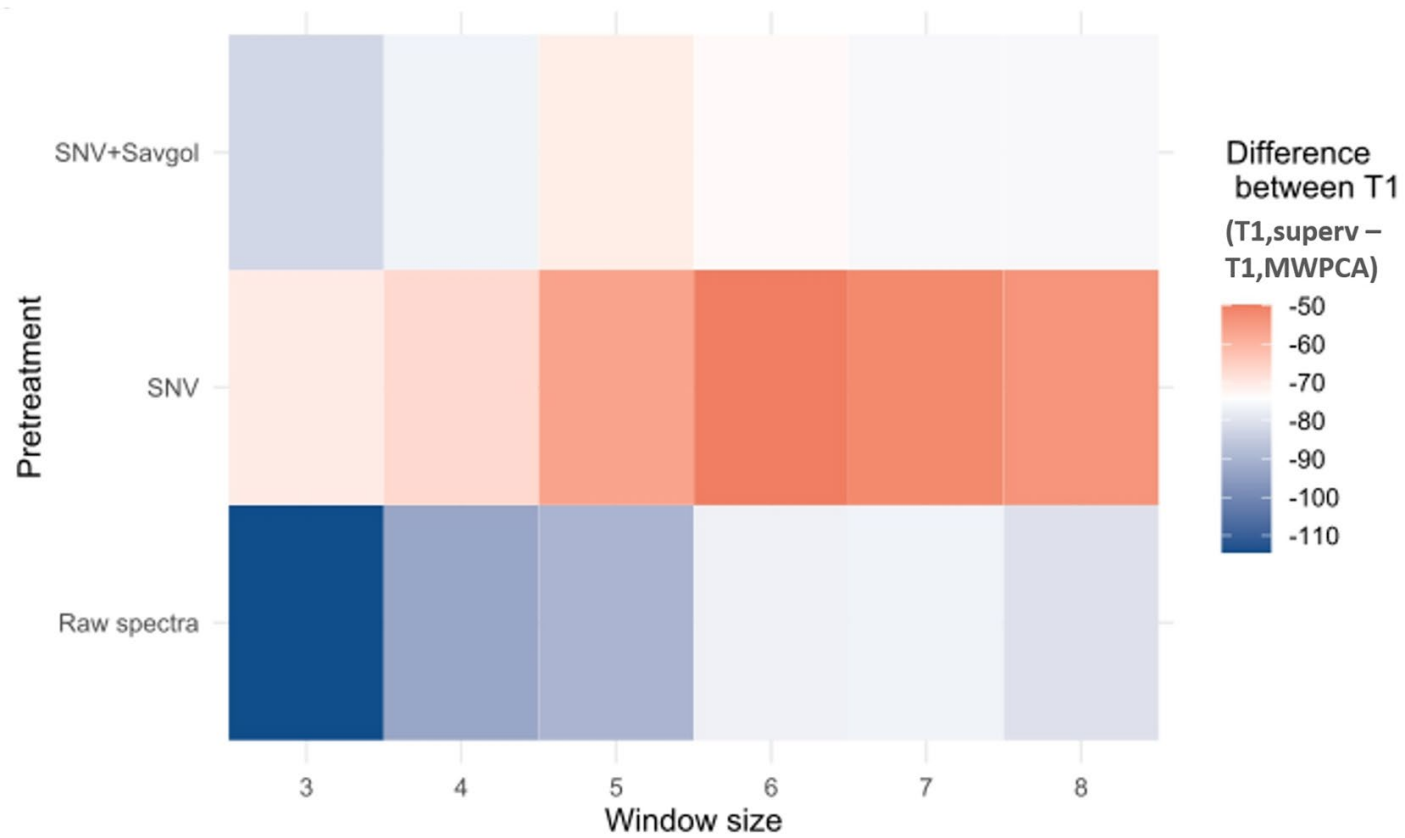
Differences expressed in GDD between the T1, superv and the T1, MWPCA according to the window size and the pretreatment

**Fig. 8:**
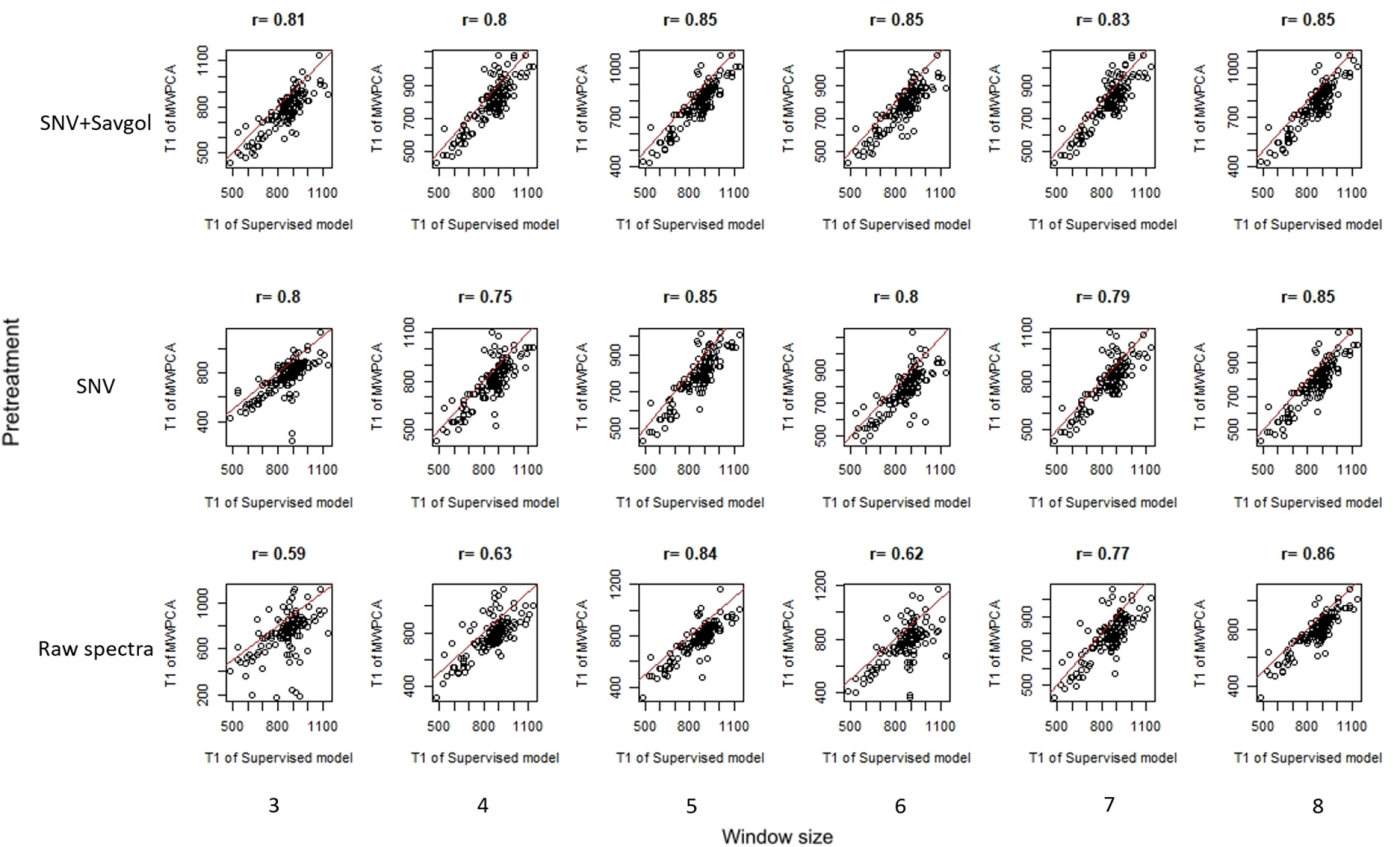
Relationship between T1, superv and T1, MWPCA. The red line corresponds to the 1:1 regression line and the number above each distribution to the correlation between the T1, superv and the T1, MWPCA

Secondly, comparisons between T1, MWPCA and T1, superv led to slightly different observations (Figs. 7 and 8). Unlike with T0, MWPCA provided earlier values for T1 compared to the supervised model (from 110 to 50 GDD). The spectra pre-treatment affected the range of these differences: it was maximal with raw spectra (~ 114 GDD) and minimal with the SNV spectra (~ 50 GDD). Regardless of the pre-treatment, increasing the window size decreased the difference between the two indicators T1, MWPCA and T1, superv.

As for T0 parameters, the use of raw spectra requires higher window size to generate correlation values similar to SNV or SNV-Savgol spectra. When looking at the distribution (Fig. 8), the point clouds show that the T1 from the MWPCA align themselves with the T1 from the supervised model, albeit slightly earlier. Unlike for T0, the point clouds are more constant in their form for all pretreatments and all numbers of components.

## Discussion

Based on fresh leaves, the calibrations obtained using spectral data sets to assess water, nitrogen or chlorophyll content are supported by existing literature [27–29, 34] and provide accurate predictions for the three leaf biochemical variables. Daily measurements on the same flag leaf were used to model the temporal kinetics of the three traits during the senescence process. The shape of the curves was similar for each parameter, and could be described by a sigmoid curve following a pattern ‘plateau/drop/plateau’ with four parameters. The pattern of these temporal dynamics is consistent with that observed for leaf senescence [4, 15].

Based on these curves, two key dates, named T0 and T1 respectively, which are related to the beginning and end of the resorption or desiccation processes, were targeted. Nitrogen fertilization coupled with genetic diversity provided a large variability range for these 2 key points (SD ≥  ± 100 °C) whatever the biochemical component similar to results previously reported [35]. On average, T0 mean values of the three-biochemical components varied significantly (mean difference = 154 GDD) whereas T1 values were quite similar (mean difference = 44 GDD).

According to the spectra pre-treatment used, from three to four T^2^ peaks characterizing some leaf spectra changes were detected by the MWPCA. Most of these changes occurred in a time course within the earliest T0, superv (most often related to chlorophyll content) and during the late T1,superv. Window size can sometimes influence the shape of the distribution (Figs. 5B and 6B). The higher number of peaks found by the MWPCA, provides more information, but this information is not always relevant. The choice of the best pretreatment method or window size cannot be based on this parameter alone.

The ability of MWPCA to detect spectra changes according to T0 or T1 computed in the supervised model was uneven. The first peak of the MWPCA was later than the earliest T0 from the supervised strategy (~ 100 GDD). Moreover, the correlation between the two data sets remained weak (r < 0.6) indicating that in our study MWPCA based on a global spectra approach is not relevant to signal senescence onset.

MWPCA performed well when used to detect the end of the senescence: the later T^2^ peak was highly correlated with the earlier T1 value obtained using a supervised strategy (up to r = 0.86) and the timing of these two key points are similar (~ 50 GDD). The last peak of the MWPCA seems to correspond to the end of the process, represented by T1.

**Fig. 9:**
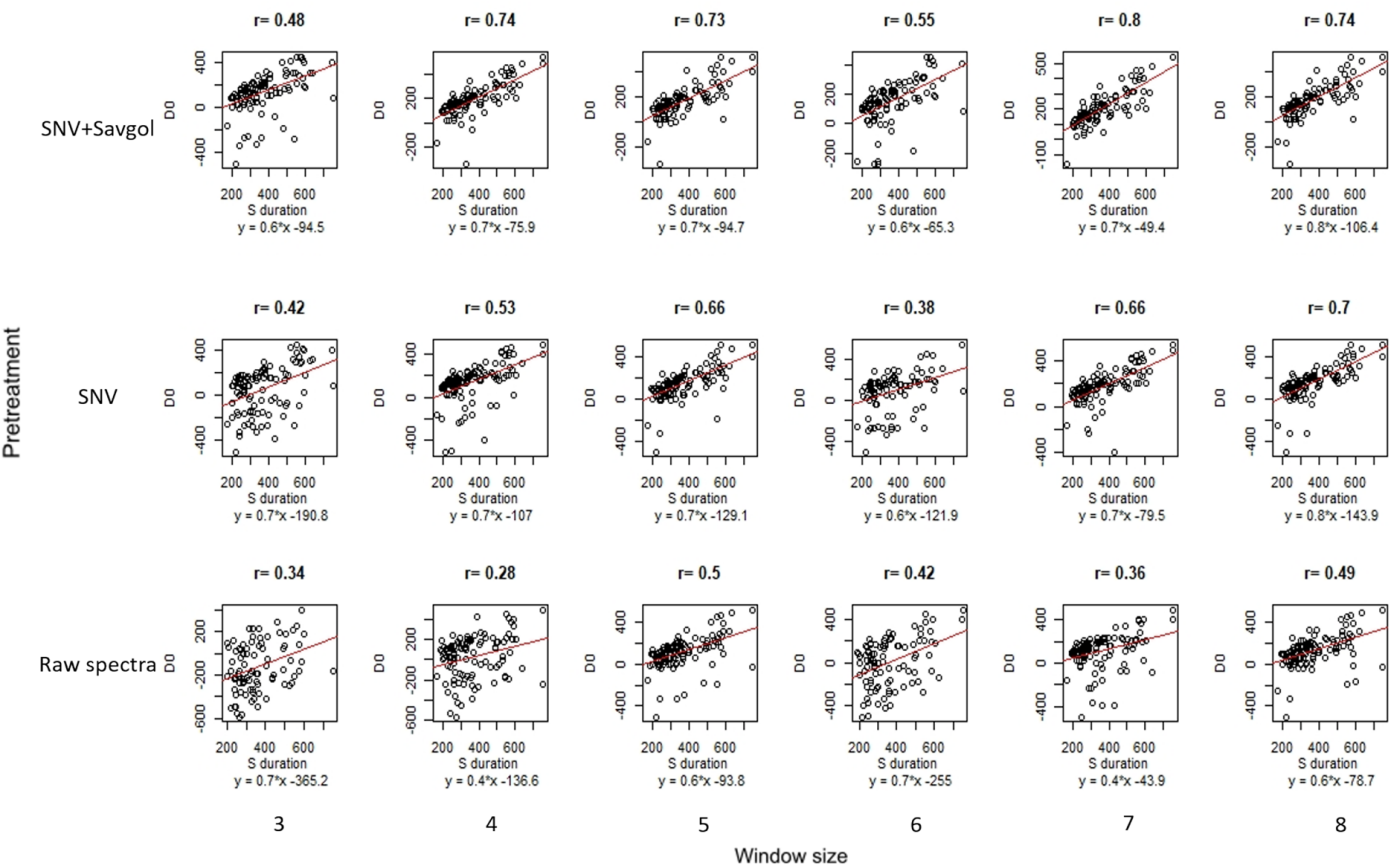
Correlation of the difference between the T0, MWPCA timing and the T0, superv timing expressed (D0) and the duration of the senescence (Sduration) in GDD according to the window size and the pretreatment. The red line corresponds to the regression line between the two parameters. The number above each distribution corresponds to the correlation between D0 and Sduration

Senescence duration, i.e. the time between T0 and T1, explains largely variances in the ability of MWPCA to predict T0 and T1. A shorter duration, i.e. a higher senescence rate, induced rapid changes in the biochemical components of leaves, which significantly modified the spectra signature. These bigger changes are better observed by the MWPCA [36] as shown by the strong correlation (up to r = 0.80, Fig. 9) between the senescence duration and the discrepancies between the T0, MWPCA and the T0, superv. These data suggest that a non-supervised strategy based on MWPCA as used here will be unable to identify some changes in spectra data if the properties of the matrix evolve too slowly. This observation is coherent with other studies conducted on others processes [37, 38].

The coupling of no spectra pre-treatment and a small learning window size downgraded the previous results. With these raw spectra data sets, a minimum of a 5-day window size was required to obtain good quality results. We assumed that it could be related to the importance of the variability of overall reflectance amplitude and base line [38]. It has been reported that SNV pretreatement reduces the variability of a spectra data set generated by physical composition heterogeneity of the samples resulting in multiplicative and additive effects [39, 40]. Indeed, differences of micro-environmental conditions from one day to the other could have resulted in heterogeneity in the physical composition of the leaves [41]. Moreover, the position of the leaf in the LeafClip could have been shifted when acquiring the spectrum, so that additive and multiplicative effect could appear. Since the senescence process is more related to biochemical than physical composition, SNV pre-treatment is an appropriate correction to use MWPCA. Reducing the spectral daily unwanted heterogeneity makes it possible to get some results that were less dependent on the window size. The minimal window size was reduced to three days instead of five previously and Savgol pre-treatment allowed us to obtain similar results whichever the window size.

Looking at the average distance between key points (T0, T1) computed from supervised and MWPCA methods, the best pretreatment is SNV, with a window size from 6 to 8. If the criterion is the correlation coefficient, the pretreatment and the size of the windows should be changed according to T0 or T1. SNV pretreatment with a minimum 5-day window size seems to be the best compromise.

Prior knowledge of the biological process involved in senescence is important when choosing some MWPCA parameters and interpreting results. For example, one important piece of information to be aware of is that all the individuals have a plateau (for the first part of their sigmoidal curve) related to at least eight measurements. This information can be used to define a priori a range of window sizes for learning. This was possible because we had previous knowledge thanks to the supervised model. This information was important for the MWPCA because it meant that the learning of the MWPCA could not be conducted inside the process that was studied. This knowledge is something that is usually known when working with the MWPCA.

## Conclusions perspectives

Our objective was to manage a complex phenotype thanks to a non-supervised model applied to whole spectra. Here leaf senescence was considered here as a use case.

Overall, our study demonstrates that the information contained in the spectra can be used when applying an unsupervised method, in this case the MWPCA, to characterize a complex biological phenomenon such as leaf span. Of course, further work is needed firstly to clarify whether the difficulties in predicting some key points of senescence such as T0 are due to the method used (i.e. MWPCA) or intrinsic to the unsupervised strategy and, secondly, to validate this method as a screening method under field or controlled environments. For this later point, reducing spectra complexity has to be considered; in relation to light absorbance in visible (pigments), NIR and SWIR regions (water, Nitrogen) we observed that reducing the spectra to the visible or to the SWIR regions (400–1000 nm and 1000–2500 nm, respectively) did not change the results obtained with the full spectral range (data not shown). These results suggest that it is possible to downgrade the device while limiting the risk to document senescence process just on a leaf colour changes. On the other hand, since some other foliar macronutrients such as S, Ca, Mg, P and K have a spectral signature on fresh individual leaves [42], it might be interesting to document the evolution of their content over the life span of the wheat leaves, such as done for water, nitrogen and chlorophyll. This would allow having a better understand of the phenomena inducing whole spectral changes. Finally, to characterise the initial state of the leaf, spectral pre-processings such as SNV or Savgol are relevant even with a reduced number of daily spectra included in the reference spectral dataset. Smaller dataset permits the use of a small training window size, which is interesting especially when the senescence process is rapid, such as under conditions of high temperature or mineral depletion.

To conclude, our work suggests that considering the whole spectra, such as that of a phenotypic trait, based on a holistic approach, to document complex biological process may be relevant for tackling both applied issues in the agricultural context, and forefront research questions in plant biology.

## Supplementary Information

**Additional file 1: Table S1.** Pretreatments and statistics of the calibration of the supervised model [24].

## Data Availability

The data that support the findings of this study are available from the corresponding author upon reasonable request.
