# Supplementary material for: Unsupervised analysis of NIRS spectra to assess complex plant traits: leaf senescence as a use case
Source: Plant Methods. 2022 Aug 12;18:100. doi: 10.1186/s13007-022-00927-6 (PMC9373489; doi:10.1186/s13007-022-00927-6)
Supplement: Supplementary file 1 — Additional file 1: Table S1. Pretreatments and statistics of the calibration of the supervised model [24]. [file 13007_2022_927_MOESM1_ESM.docx]

**Supplementary material**

For all calibrations, spectra were collected on fresh leaves, at different stages between anthesis and maturity, that were representative of the genotypic diversity used in this study.

Leaf Nitrogen Content (LNC): The complete description of the method is presented in Ecarnot et al., 2013.

Leaf Chlorophyll Content (LCC) : Chlorophyll reference analysis was done as follows: Less than one hour after collecting spectra, approx. 20 mg of leaves were cut (the area where the spectra had been taken), and stored at -20 °C. After grinding, powder is diluted into ethanol and optical density at wavelengths 645 and 665 is measured by a SAFAS Xenuis spectrofluorometer, in order to estimate chlorophylls a and b. In this study, LCC is the sum of chlorophylls a and b in mg/g of fresh matter.

Leaf water Content (LWC): fresh leaf weight (FW) is measured just after collecting spectra, the leaves are dried 48 hours at 60°C, then dry weight (DW) is measured. LWC is computed as follows: LWC = 100 x (FW-DW) / FW

To develop calibrations, different pretreatments were tested: Standard Normal Variate transformation (SNV, Barnes et al., 1989), reduction of wavelengths at the ends of spectra (RED), or Savitzky–Golay derivation (SG, Savitzky and Golay, 1964). The best combination of pretreatment was kept on the basis of the optimization of the standard error of cross validation (SECV) (minimize it with a minimum of latent variables).

SG parameters refer, resp., to the degree of the fitting polynomial, the size of the fitting window, and the order of derivation.

SECV and the coefficient of determination R2CV are both computed for each latent variable with following formulas:

$$SECV = \sqrt{\sum_{\frac{{}}{n}} (yi - \hat{yi}){}^{2}}$$

$$R^{2}CV =\left( \frac{\sum_{i}^{n} (yi - \underline{yi})\times(\hat{y}i -\hat{\underline{yi}})}{\sigma(y)\times\sigma(\underline{y})} \right)^{2}$$

where y is the parameter from the reference method, and $\hat{y}$ is the parameter computed in the validation step of the cross-validation

Pretreatments and statistics of the calibrations are summed up in the following table

Table S1: Pretreatments and statistics of the calibration of the supervised model [24]

|  | Number of sample for calibration | Range | Pretreatment | Number of latent variable | SECV | R^2^CV |
| --- | --- | --- | --- | --- | --- | --- |
| Nitrogen content (% DM) | 359 | 0.48–4.85 | SNV + SG[2-9-1] | 12 | 0.372 | 0.918 |
| Chlorophyll Content (mg/g FM) | 128 | 0.15-3.86 | RED[450-1350] + SNV + SG[2-9-1] | 5 | 0.249 | 0.927 |
| Water Content (%) | 262 | 1.79 - 77.54 | SNV + SG[2-3-1] | 5 | 6.47 | 0.952 |
